# Health disparity and mortality trends of infectious diseases in BRICS from 1990 to 2019

**DOI:** 10.7189/jogh.12.04028

**Published:** 2022-03-26

**Authors:** Qiao Liu, Wenzhan Jing, Min Liu, Jue Liu

**Affiliations:** 1 Department of Epidemiology and Biostatistics, School of Public Health, Peking University, Beijing, China; 2 Institute for Global Health and Development, Peking University, Beijing, China

## Abstract

**Background:**

Brazil, Russia, India, China, and South Africa (BRICS) represented almost half of the global population and much infectious disease burden. We aimed to analyze the current status and trends from 1990 to 2019 of infectious disease mortality in BRICS.

**Methods:**

We used the data of mortality estimation from the Global Burden of Disease Study 2019. The absolute number of deaths from and mortality rates of infectious diseases in each country were derived from the database from 1990 to 2019. Age-standardized mortality rate (ASMR) was used to compare populations in different regions and times. The estimated annual percentage change (EAPC) of rates quantified the infectious disease mortality trends.

**Results:**

BRICS respectively accounted for 39% and 32% of the global infectious disease deaths, in 1990 and 2019. Lower respiratory infections, tuberculosis, and diarrheal diseases contributed the most to the number of deaths in 1990 and 2019. In BRICS, ASMRs of all infectious diseases except sexually transmitted infections (STIs) decreased. The highest STI ASMRs were in South Africa; the highest ASMRs of enteric infections, neglected tropical diseases and malaria, and other infectious diseases were in India; South Africa and India both had relatively high respiratory infection ASMRs.

**Conclusion:**

Infectious disease mortality varies substantially in BRICS, and health disparity needs to be considered when facing complex infectious disease situations in different countries.

BRICS – Brazil, Russia, India, China, and South Africa – represented more than 40% of the world’s population, around 25% of the world’s gross national income, about 50% of the world’s poor, and about 40% of the global burden of disease [1,2]. During the coronavirus disease 2019 (COVID-19) pandemic, BRICS together accounted for almost 30% of all deaths caused by COVID-19 across the world [3,4]. Previous studies had shown that certain infectious diseases were a great burden in BRICS. South Africa was considered to have the largest number of people infected with human immunodeficiency virus (HIV) and acquired immunodeficiency syndrome (AIDS) deaths in the world [5]. BRICS accounted for more than 30% of the world’s children at risk of infection with soil-transmitted helminths [6]; they also bore 49% of the world’s burden of tuberculosis (TB), 40% of all TB-related mortality, and more than 60% of the multidrug-resistant TB burden [7]. India also experienced the world’s largest absolute burden of at least 11 major neglected tropical diseases [8]. Moreover, India and China had the second- and third-highest ASMR of Hepatitis E virus infection [9].

The Sustainable Development Goals (SDGs) aimed to end the epidemics of AIDS, TB, malaria, and neglected tropical diseases (NTDs), and combat hepatitis, waterborne diseases, and other communicable diseases, all by 2030 [10]. Despite the decrease in mortality and morbidity from major infectious diseases, the world is not on track to meet the 2020 milestones of SGDs of certain infectious diseases, such as malaria and TB [11,12]. To achieve the goals of SDGs, huge efforts need to be taken by countries all over the world, especially for BRICS. BRICS were well positioned to exert a significant influence on global health, not only because they accounted for nearly half the world’s population, but also because they served as important role models for other countries within their respective regions [13].

However, there is a lack of studies which could provide a comprehensive description of various infectious diseases in BRICS. Therefore in this study, we analyzed the current status and trends from 1990 to 2019 of infectious disease mortality in BRICS, using the data from Global Burden of Disease Study 2019 (GBD 2019) results [14]. Our study can provide a comprehensive perspective for understanding the long-term trends and regional differences in mortality from various infectious diseases among BRICS. A better understanding of infectious disease mortality could make global strategies to eliminate infectious diseases more scientific and sounder.

## METHODS

### Data Sources

We used data obtained in the GBD 2019, which consisted of a systematic and scientific effort to quantify the comparative magnitude of health losses due to diseases by sex, age, and location over time [15]. From the website named “Global Health Data Exchange”, established by the GBD group [14], we extracted the annual number of deaths from and mortality rates of 5 categories of infectious diseases in BRICS from 1990 to 2019, by sex and age. These 5 categories of infectious diseases are: 1) HIV/AIDS and sexually transmitted infections (STIs), 2) respiratory infections and TB, 3) enteric infections, 4) NTDs and malaria, and 5) other infectious diseases. The general methodological approaches for estimating mortality were described elsewhere [16]. In short, all available data on causes of death were standardized and pooled into a single database used to generate cause-specific mortality estimates by age, sex, year, and geography; then, multiple models were used to estimate comparable mortality data of different diseases across the world [16].

We reported the mortality from 5 categories of infectious diseases in Brazil, Russia, India, China, and South Africa (BRICS) from 1990 to 2019 and arranged mortality data into successive 5-year age intervals form 0-4 years to 80-84 years.

### Statistical Analyses

The absolute number of deaths represented the actual condition of infectious diseases in each country, and its relative change (%) was defined as:

[(Number of deaths_2019_ – Number of deaths_1990_)/Number of deaths_1990_] × 100

which showed the overall difference between 1990 and 2019. Age-standardized mortality rate (ASMR) was calculated by applying the age-specific rates to a GBD World Standard Population. ASMRs could be extracted directly from the website [14], and were used to compare populations with different age structures or the same population over time in which the age profiles change accordingly.

Estimated annual percentage change (EAPC) was widely used to quantify the rate trend over a specific interval. A regression line was fitted to the natural logarithm of the rates (*γ = α + β_x_ + ε*)where *y* = ln (rate) and *x* = calendar year). EAPC was calculated as 100 × (*e^β^* – 1), with 95% confidence intervals (CIs) obtained from the linear regression model. In this study, overall EAPC was calculated from the annual ASMR of each category of infectious diseases in BRICS, and EAPC in different age groups was calculated from the age-specific mortality rate. The term “increase” was used to describe trends when the EAPC and its lower boundary of 95% CI were both >0. In contrast, “decrease” was used when the EAPC and its upper boundary of 95% CI were both <0. Otherwise, the term “stable” was used.

## RESULTS

### Number of deaths from leading infectious diseases in BRICS

**Figure 1:**
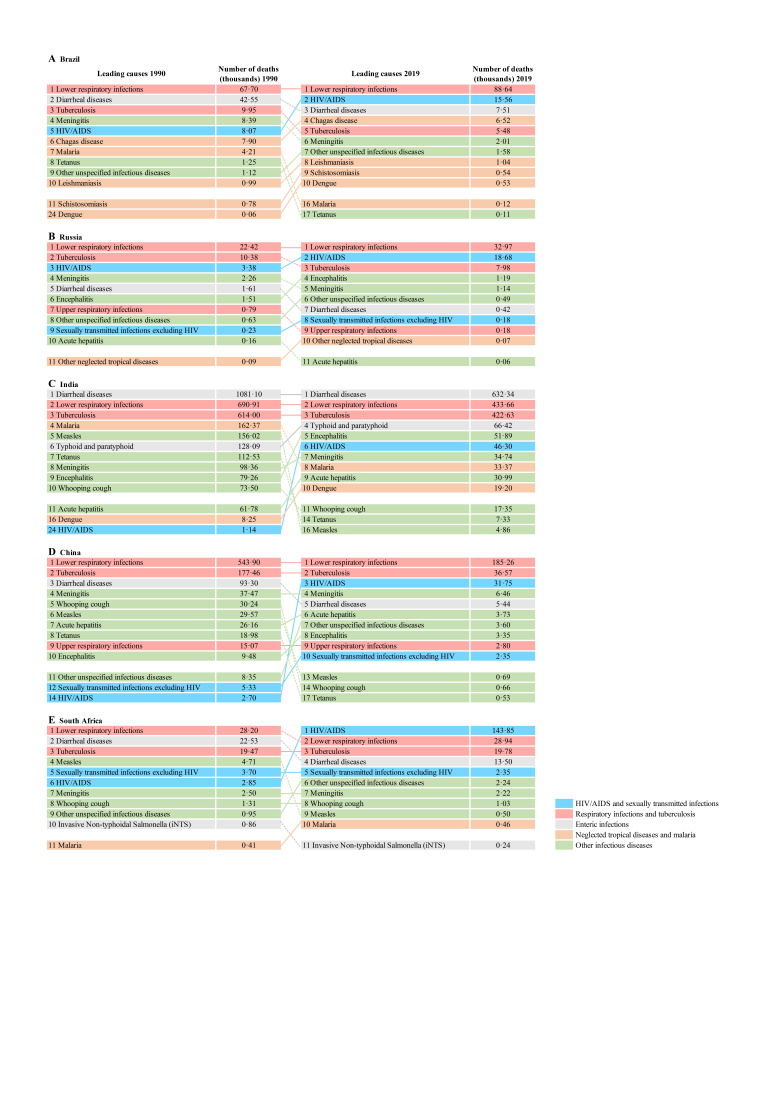
Leading 10 infectious diseases of number of deaths in BRICS (1990 and 2019). Diseases are connected by lines between time periods; solid lines are increases in rank and dashed lines are decreases.

As shown in **Figure 1**, the distribution of infectious disease mortality varied greatly in BRICS. Generally speaking, lower respiratory infections (LRIs), TB, and diarrheal diseases contributed the largest number of deaths in 1990 and 2019; and HIV/AIDS also became a huge threat to health in 2019. LRIs caused the most deaths in Brazil, Russia, and China in both 1990 and 2019. India suffered the most from diarrheal diseases, and 632 340 people died in 2019. The number of deaths from LRIs was the second highest, with 433 660 deaths in 2019. In South Africa, deaths resulting from HIV/AIDS increased from 2850 in 1990 (sixth highest) to 143 850 in 2019 (the highest), much more than the second highest – LRIs, with 28 940 deaths in 2019. Although the fifth kind of infectious diseases (other infectious diseases) did not cause as many deaths as other 4 kinds of infectious diseases. Certain diseases such as meningitis, encephalitis, and acute hepatitis were also threating people’s lives.

### Time trends in mortality of infectious diseases

**Figure 2:**
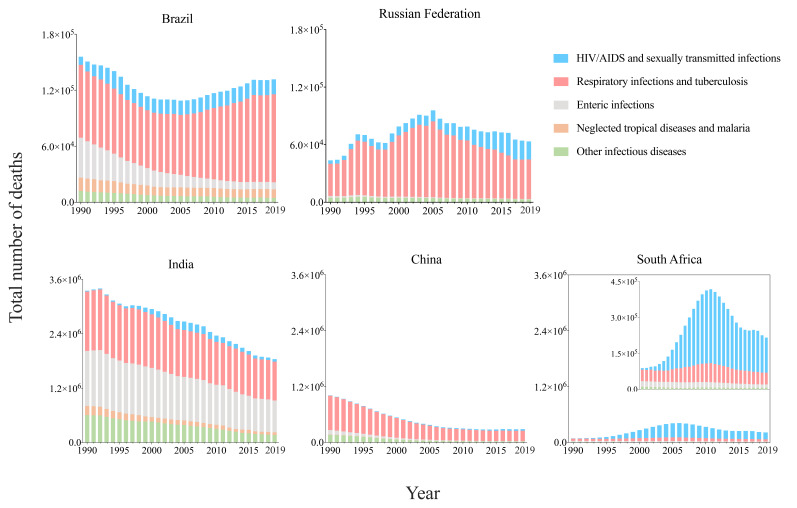
The number of infectious diseases by Brazil, Russia, India, China, and South Africa, from 1990 to 2019. HIV/AIDS - human immunodeficiency virus/Acquired Immune Deficiency Syndrome.

In BRICS, there were 4.65 million deaths resulting from infectious diseases in 1990, and 2.54 million deaths in 2019, respectively accounting for 39% and 32% of the global deaths resulting from infectious diseases (**Table 1**). The number of deaths decreased by 45.39% in BRICS, higher than the global decrease of 34.09%. Deaths resulting from respiratory infections and TB accounted for a relatively large proportion in Brazil, Russia, India, and China. The number of deaths from enteric infections was close to that from respiratory infections and TB in India. In South Africa, the number of deaths from HIV/AIDS and STIs was much higher than other 4 categories of infectious diseases (**Figure 2**).

**Table 1:** The number of deaths and age-standardized mortality rates of infectious diseases in BRICS in 1990 and 2019, and their temporal trends from 1990 to 2019

	Age-standardized mortality rate			Number (thousands)					
	**1990**	**2019**	**EAPC (%) (95% CI)**	**1990**		**2019**		**Change (%)**	
**Overall infectious diseases**									
Global	–	–	–	11 930.60		7862.91		-34.09	
BRICS	–	–	–	4649.43		2539.05		-45.39	
BRICS/Global	–	–	–	0.39		0.32		–	
**HIV/AIDS and sexually transmitted infections**									
Global	8.07 (6.04 to 10.72)	12.08 (10.64 to 14.22)	0.06 (-1.52 to 1.66)	442.91 (327.51 to 589.5)		953.73 (849.27 to 1108.38)		115.33 (81.26 to 165.07)	
Brazil	6.09 (5.98 to 6.20)	6.63 (6.52 to 6.74)	-1.08 (-1.64 to -0.52)	8.69 (8.52 to 8.87)		16.01 (15.73 to 16.27)		84.13 (79.28 to 88.73)	
Russia	2.30 (2.27 to 2.32)	11.48 (11.36 to 11.60)	5.91 (5.4 to 6.41)	3.6 (3.57 to 3.64)		18.86 (18.66 to 19.07)		423.36 (416.35 to 430.91)	
India	1.77 (0.89 to 3.37)	4.09 (3.50 to 4.99)	3.14 (0.11 to 6.26)	19.11 (8.19 to 38.8)		55.89 (48.41 to 67.37)		192.52 (64.58 to 526.72)	
China	0.73 (0.41 to 1.21)	2.02 (1.63 to 2.43)	3.38 (3.13 to 3.62)	8.03 (4.41 to 13.6)		34.1 (27.84 to 40.13)		324.59 (154.23 to 711.68)	
South Africa	16.06 (9.96 to 24.61)	255.65 (224.32 to 307.10)	8.01 (4.26 to 11.9)	6.55 (3.97 to 10.29)		146.2 (126.38 to 179.91)		2131.44 (1336.99 to 3478.07)	
BRICS/Global	–	–	–	0.10		0.28		–	
**Respiratory infections and tuberculosis**									
Global	107.53 (100.53 to 114.70)	49.09 (44.94 to 53.66)	-2.82 (-2.90 to -2.74)	5135.02 (4752.4 to 5569.73)		3683.1 (3382.55 to 4011.67)		-28.27 (-35.23 to -20.57)	
Brazil	74.57 (69.65 to 80.55)	43.47 (38.33 to 46.26)	-1.54 (-1.9 to -1.19)	78.09 (72.4 to 86.54)		94.26 (83.47 to 100.23)		20.71 (4.99 to 33.32)	
Russia	23.26 (22.38 to 24.07)	20.74 (18.10 to 23.67)	-1 (-1.97 to -0.02)	33.59 (32.3 to 34.56)		41.13 (35.76 to 47.27)		22.44 (6.87 to 40.28)	
India	212.86 (190.80 to 233.62)	79.73 (70.34 to 90.67)	-3.53 (-3.66 to -3.4)	1309.49 (1177.83 to 1438.36)		857.09 (753.94 to 976.65)		-34.55 (-43.72 to -24.35)	
China	84.59 (76.23 to 91.67)	15.99 (13.97 to 18.40)	-6.18 (-6.38 to -5.98)	736.61 (655.73 to 808.21)		224.65 (194.33 to 259.86)		-69.5 (-74.31 to -61.43)	
South Africa	164.64 (147.25 to 180.68)	110.32 (101.84 to 118.72)	-0.98 (-1.69 to -0.26)	47.93 (43.5 to 52.29)		48.88 (45.18 to 52.9)		1.97 (-7.95 to 12.48)	
BRICS/Global	–	–	–	0.43		0.34		–	
**Enteric infections**									
Global	62.88 (47.84 to 80.58)	23.95 (17.76 to 32.45)	-3.34 (-3.46 to -3.23)	3166.78 (2500.76 to 3901.53)		1748.25 (1286.41 to 2416.19)		-44.79 (-56.4 to -26.74)	
Brazil	30.29 (26.42 to 35.77)	3.81 (3.39 to 4.21)	-7.41 (-7.72 to -7.1)	42.74 (36.64 to 51.8)		7.59 (6.74 to 8.26)		-82.25 (-85.72 to -78.77)	
Russia	1.39 (1.33 to 1.48)	0.29 (0.26 to 0.33)	-6.83 (-7.48 to -6.18)	1.65 (1.58 to 1.74)		0.45 (0.4 to 0.51)		-72.8 (-75.97 to -69.46)	
India	285.72 (187.61 to 389.36)	76.26 (45.05 to 121.63)	-4.64 (-4.84 to -4.44)	1213.64 (871.95 to 1592.59)		701.67 (419.72 to 1121.82)		-42.18 (-59.07 to -15.4)	
China	9.25 (7.54 to 11.36)	0.55 (0.42 to 0.76)	-10.38 (-10.84 to -9.92)	96.72 (80.96 to 115.17)		6.68 (4.89 to 10.04)		-93.09 (-94.93 to -90.05)	
South Africa	71.98 (53.11 to 101.07)	32.52 (21.35 to 55.01)	-2.65 (-3.04 to -2.25)	23.42 (18.16 to 31.34)		13.76 (9.48 to 22.42)		-41.24 (-54.97 to -21.7)	
BRICS/Global	–	–	–	0.44		0.42		–	
**Neglected tropical diseases and malaria**									
Global	18.06 (11.26 to 27.02)	10.32 (5.61 to 17.27)	-1.89 (-2.28 to -1.49)	1034.12 (638.52 to 1551.48)		747.34 (406.56 to 1247.14)		-27.73 (-53.36 to 5.31)	
Brazil	13.18 (6.61 to 17.51)	4.03 (2.16 to 6.06)	-3.99 (-4.28 to -3.7)	14.38 (7.48 to 20.01)		9.14 (4.99 to 13.61)		-36.46 (-55.93 to 25.76)	
Russia	0.11 (0.07 to 0.14)	0.06 (0.04 to 0.08)	-2.49 (-2.88 to -2.09)	0.16 (0.11 to 0.21)		0.1 (0.06 to 0.13)		-37.59 (-53.16 to -18.03)	
India	21.03 (10.68 to 41.97)	5.06 (2.92 to 9.02)	-4.82 (-5.2 to -4.43)	203.96 (100.43 to 404.88)		61.87 (35.11 to 112.62)		-69.66 (-84.41 to -42.07)	
China	0.67 (0.46 to 2.10)	0.11 (0.08 to 0.13)	-5.77 (-6.42 to -5.11)	6.68 (4.37 to 23.51)		1.86 (1.31 to 2.22)		-72.14 (-92.21 to -56.49)	
South Africa	1.54 (0.45 to 4.51)	0.97 (0.17 to 7.22)	-6.2 (-7.82 to -4.55)	0.54 (0.15 to 1.61)		0.54 (0.09 to 4.06)		-0.05 (-92.81 to 977.53)	
BRICS/Global	–	–	–	0.22		0.10		–	
**Other infectious diseases**									
Global	36.25 (27.42 to 50.79)	10.19 (8.33 to 12.79)	-4.57 (-4.78 to -4.36)	2151.77 (1584.26 to 3062.21)		730.49 (605.26 to 904.68)		-66.05 (-73.48 to -57.68)	
Brazil	8.16 (7.41 to 9.02)	2.31 (2.04 to 2.71)	-4.37 (-4.56 to -4.19)	12.07 (10.91 to 13.46)		4.75 (4.22 to 5.62)		-60.68 (-66.35 to -53.17)	
Russia	3.53 (3.08 to 3.83)	1.92 (1.55 to 2.25)	-2.68 (-3.03 to -2.33)	4.73 (4.26 to 5.06)		2.93 (2.5 to 3.38)		-38.12 (-45.99 to -28.28)	
India	62.68 (50.47 to 80.65)	14.13 (11.57 to 17.53)	-5.08 (-5.39 to -4.76)		603.72 (459.57 to 812.95)		164.66 (133.51 to 205.44)		-72.73 (-80.27 to -62.46)	
China	15.13 (11.77 to 20.55)	1.51 (1.30 to 1.76)	-8.42 (-8.83 to -8.01)	163.24 (124.54 to 226.39)		19.59 (16.96 to 22.38)		-88 (-91.53 to -83.83)	
South Africa	24.84 (17.38 to 36.26)	13.03 (10.14 to 16.79)	-1.52 (-2 to -1.04)	10.09 (6.71 to 15.29)		6.39 (4.88 to 8.3)		-36.61 (-57.17 to -4.34)	
BRICS/Global	–	–	–	0.37		0.27		–	

BRIC – Brazil, Russia, India, China, and South Africa, EAPC – estimated annual percentage change, HIV/AIDS – human immunodeficiency virus/Acquired Immune Deficiency Syndrome, CI – confidence interval

**Figure 3:**
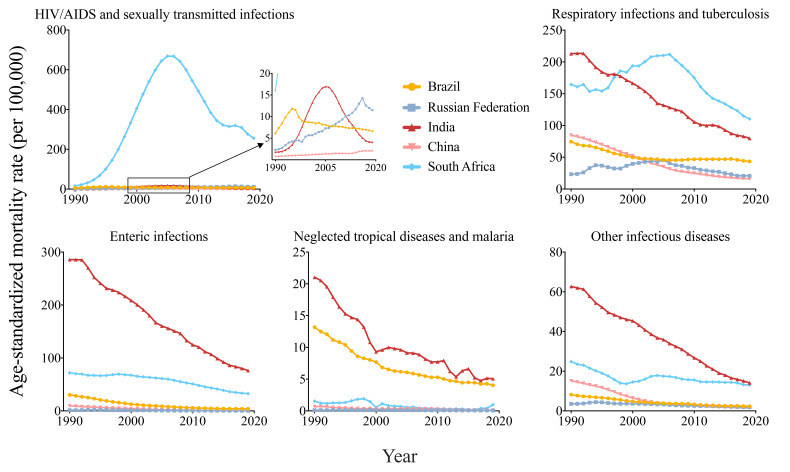
Age-standardized mortality rates of infectious diseases in Brazil, Russia, India, China, and South Africa, from 1990 to 2019. HIV/AIDS - human immunodeficiency virus/Acquired Immune Deficiency Syndrome.

The ASMR of HIV/AIDS and STIs increased the fastest in South Africa by an average 8.01% (95% CI = 4.26-11.9) per year from 16.06 per 100 000 in 1990 to 255.65 per 100 000 in 2019 (**Table 1**). The ASMR of HIV/AIDS and STIs was much higher in South Africa than in other 4 countries (**Figure 3**). From 1990 to 2019, there was a trend of an increase followed by a decrease in ASMRs of HIV/AIDS and STIs in Brazil, Russia, India, and South Africa, with different inflection points (Brazil, 11.81 per 100 000 in 1995; Russia, 14.25 per 100 000 in 2016; India, 16.94 per 100 000 in 2005; South Africa, 668.30 per 100 000 in 2005). Brazil was the only country with a decreasing ASMR of HIV/AIDS and STIs (EAPC = -1.08, 95% CI = -1.64 to -0.52). However, in China, the ASMR of HIV/AIDS and STIs continuously increased from 0.73 per 100 000 in 1990 to 2.02 per 100 000 in 2019 (EAPC = 3.38, 95% CI = 3.13-3.62).

For respiratory infections and TB, ASMRs in India and South Africa were higher in the other 3 countries (**Figure 3**). Brazil (EAPC = -1.54, 95% CI = -1.90 to -1.19), India (EAPC = -3.53, 95% CI = -3.66 to -3.40) and China (EAPC = -6.18, 95% CI = -6.38 to -5.98) had continuously decreasing ASMRs from 1990 to 2019, while Russia (EAPC = -1.00, 95% CI = -1.97 to -0.02) and South Africa (EAPC = -0.98, 95% CI = -1.69 to -0.26) had a trend of an increase followed by ad decrease. In 1990, India had the highest ASMR (212.86 per 100 000), followed by South Africa (164.64 per 100 000). In 2019, South Africa became the country with the highest ASMR (110.32 per 100 000), followed by India (79.73 per 100 000).

All ASMRs of enteric infections, NTDs and malaria, and other infectious diseases in BRICS had overall downtrends from 1990 to 2019; however, there were sometimes uptrends, such as ASMRs of NTDs and malaria in India in 2000-2003 and 2013-2015, and in South Africa from 2015 to 2019. For these 3 kinds of infectious diseases, India had the highest ASMRs. (**Figure 3**).

### Age-specific mortality rates of infectious diseases

**Figure 4:**
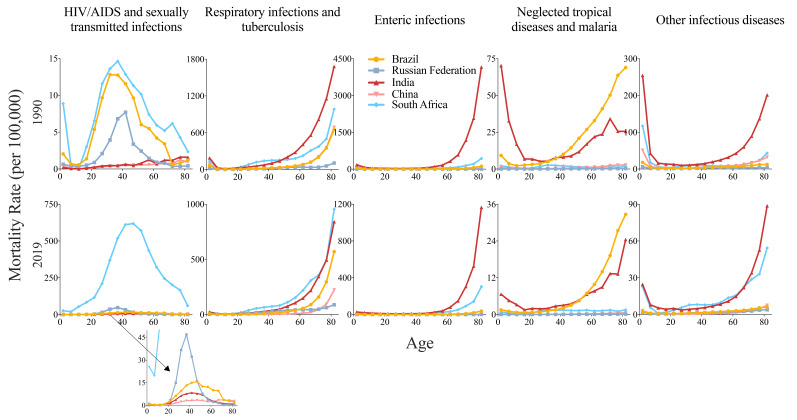
Age-specific mortality rates of infectious diseases in Brazil, Russia, India, China, and South Africa, in 1990 and 2019. HIV/AIDS - human immunodeficiency virus/Acquired Immune Deficiency Syndrome.

In 2019, the highest mortality rates of HIV/AIDS and STIs were observed in the age groups of 35-39 years in Russia (47.01 per 100 000), the 40-44 years group in India (8.43 per 100 000), and the 45-49 years group in Brazil (15.93 per 100 000) and South Africa (618.32 per 100 000). Specially, there were 2 age groups in China with relatively high mortality rates, the 45-49 years group (3.56 per 100 000) and the 70-74 years group (3.72 per 100 000). In 1990, mortality gaps among BRICS were small in different age groups; however, in 2019, there was a huge gap of mortality rates of HIV/AIDS and STIs in all age groups between South Africa and other 4 countries (**Figure 4**).

For respiratory and enteric infections, there were increased mortality rates with age groups in BRICS (**Figure 4**). The highest mortality rate of respiratory infections and TB was in the 80-84 years group (1681.46 per 100 000 in India in 1990, and 955.12 per 100 000 in South Africa in 2019). For enteric infections, the highest mortality rate was 4162.20 per 100 000 in 1990 and 1165.83 per 100 000 in 2019, both in India.

For NTDs and malaria, and other infectious diseases, relatively high mortality rates were observed in both younger and older groups (**Figure 4**). Mortality rates of NTDs and malaria were higher in Brazil and India (32.68, 24.49 per 100 000, in 2019, 80-84 years group), and mortality rates of other infectious diseases were higher in India and South Africa (88.74, 54.32 per 100 000, in 2019, 80-84 years group).

### EAPC in different age groups

**Figure 5:**
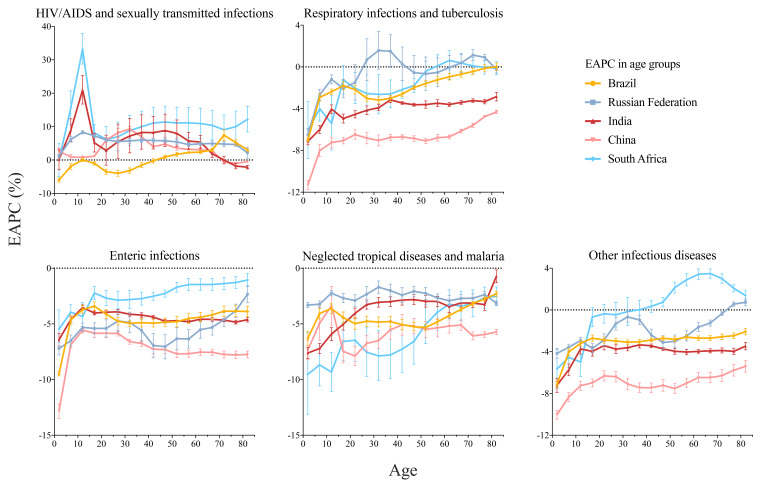
EAPC in different age groups of infectious diseases in Brazil, Russia, India, China, and South Africa and the corresponding 95% CIs, from 1990 to 2019. EAPC, estimated annual percentage change; HIV/AIDS - human immunodeficiency virus/Acquired Immune Deficiency Syndrome.

As **Figure 5** showed, although Brazil was the only country among BRICS which showed an overall decreased ASMR of HIV/AIDS and STIs between 1990 and 2019, people aged 45 and older were facing increasing mortality rates, with the fastest increase in the 70-74 years group (EAPC = 7.42%, 95% CI = 5.85-9.03). Mortality rates of HIV/AIDS and STIs increased the most in the 10-14 years group in South Africa (EAPC = 33.21%, 95% CI = 28.72-37.85), India (EAPC = 20.97%, 95% CI = 16.76-25.32), and Russia (EAPC = 8.28%, 95% CI = 7.82-8.75). The situation in China was different, people at the age of 30-34 had the most speedily increased mortality rate (EAPC = 9.29%, 95% CI = 8.49-10.10).

For respiratory infections and TB, enteric infections, NTDs and malaria, and other infectious diseases, EPAC lay predominantly below 0 for most age groups for BRICS. However, in Russia, mortality rates of respiratory infections for the 70-74 (EAPC = 1.14%, 95% CI = 0.62-1.66) and the 75-79 (EAPC = 0.92%, 95% CI = 0.65-1.19) years groups increased, and mortality rates of other infectious diseases for the 75-79 (EAPC = 0.56%, 95% CI = 0.35-0.77) and the 80-84 (EAPC = 0.73%, 95% CI = 0.40-1.06) years group increased as well. In South Africa, people aged 45 and older had increased mortality rates of other infectious diseases, with the speediest increase in the 65-69 years group (EAPC = 3.49%, 95% CI = 3.05-3.93).

## DISCUSSION

To the best of our knowledge, this is the first comprehensive effort to summarize the state of infectious disease mortality in BRICS using data from the GBD 2019, assessing overall and age-specific mortality rates of infectious diseases, as well as their long-term trends and differences among BRICS. In 2019, the number of deaths resulting from infectious diseases in BRICS accounted for almost one third of the world’s total number, less than the proportion in 1990. Despite the decreasing mortality trends of most infectious diseases between 1990 and 2019, both absolute number of deaths from and mortality rates of HIV/AIDS and STIs increased in BRICS, and South Africa suffered the severest threat from HIV/AIDS and STIs. Meanwhile, mortality from respiratory infections and TB was relatively high in each country, compared with other kinds of infectious diseases. Age disparity was high in mortality from different infectious diseases: mortality rates were higher in middle aged groups for HIV/AIDS and STIs; for respiratory infections and TB, enteric infections, NTDs and malaria, and other infectious diseases, mortality rates increased with age. Facing with this complex situation of infectious diseases in BRICS, massive concerted and coordinated action should be taken to control mortality and reduce losses in multiple areas.

Our results showed that BRICS together accounted for a relatively high proportion of the world’s burden of infectious diseases, thus making BRICS exert a significant influence on global health. On the one hand, BRICS could directly change the global infectious disease burden by dealing with their own health problems. On the other hand, BRICS served as important role models for other countries within their respective regions, thereby indirectly influencing global health. Moreover, BRICS countries have been providing concrete assistance to low- and middle-income countries, and Brazil, China and India are in the leading place of manufacturing of low-cost medicines and vaccines [17], from which other low- and middle-income countries can also draw valuable examples. Due to the health disparity from infectious diseases among BRICS, their experience in solving the complex problems of infectious diseases could also inspire other countries with similar circumstances.

### HIV/AIDS and STIs

In China, mortality of HIV/AIDS and STIs increased steadily from 1990 to 2019, and our results showed most deaths were caused by HIV/AIDS in recent years. In the present study, middle- and older-aged population in China both showed relatively high HIV/AIDS and STI mortality rates, while the other 4 countries only showed high mortality rates among sexually active population. Recent data in China showed that HIV transmission was gradually shifting away from being caused by injecting drug use and more by sexual contact, and the proportion of HIV infections associated with sexual transmission exceeded that of injecting drug use in 2007 [18]. During the mid-1990s, the massive outbreaks of HIV infection that occurred in central China among paid plasma donors led to a huge demand for HIV/AIDS treatment and care services as more and more people became ill and died. In 2003, the Chinese government announced the “Four Frees and One Care” policy to fight against HIV/AIDS, which resulted in a significant increase in the number of people being tested for HIV and AIDS patients receiving free anti-retroviral treatment (ART) [19,20]. Historical reasons and popularity of free ART could partly explain the increased mortality rates in the elderly in China.

South Africa not only had the largest number of deaths from and highest mortality rates of HIV/AIDS and STIs (HIV/AIDS accounted for over 95%) among BRICS as our results showed, but also was considered to have the largest number of people infected with HIV and AIDS deaths all over the world [5]. Although the number of deaths from and ASMR of HIV/AIDS began to decline from 2005, HIV/AIDS was still the leading single cause of death in South Africa at an estimated speed of more than 560 deaths per day [21]. The extremely high HIV/AIDS mortality rate in South Africa might result from the high HIV/AIDS prevalence and low coverage of ART. Partner violence and the highest rape and child rape incidence in the world could increase the HIV prevalence in South Africa [22,23]. HIV prevalence among female sex workers in South Africa was estimated to be as high as 59.6%, with an estimated 131 000-182 000 sex workers in South Africa [24,25]. However, in South Africa, only 56% of people living with HIV were on ART in 2018 [26]. In recent years, it was a worthy recognition that the fight against HIV/AIDS in South Africa was entering a new era, thanks to the the largest antiretroviral treatment program in the world, a national counselling and testing campaign, promotion of medical male circumcision, and the successful implementation of a mother-to-child transmission prevention programme [27].

### Respiratory infections and TB

Despite noteworthy decreases in the mortality from respiratory infections and TB from 1990 to 2019 in BRICS, our results also showed relatively high mortality rates of respiratory infections and TB in all BRICS countries, compared with other kinds of infectious diseases.

For Brazil, Russia, and China, most deaths from respiratory infections were caused by LRIs. LRIs are also a leading cause of mortality and morbidity worldwide, disproportionately affecting children under 5 and the impoverished across the globe [28]. Pneumococcal pneumonia was the leading cause of LRI mortality globally [29]. Risk for contracting or dying from LRIs included inconsistent and insufficient access to adequate nutrition, clean cooking fuel, vaccines, and sanitation, or under immunocompromising conditions [29]. There remains an urgent need to accelerate efforts to reduce the burden of disease in the most susceptible populations. Meanwhile, in Russia, we found increased mortality rates of respiratory infections among the 70-79 years groups from 1990 to 2019. Under the combined effect of increased mortality rate and population aging, there is growing need for Russia to focus on elderly populations, while much of the global initiative to reduce the LRI burden has been focused on children under the age of 5 [29].

In India and South Africa, TB was also a huge threat to people’s health besides LRIs. India had the highest TB burden among the countries of the world, with challenges of delayed detection and treatment of TB, inadequate surveillance, poor notification, and absence of coordination with the private health care sector [11]. Though the total deaths from TB had decreased since 2007, rapid increases in death rates had been observed for extensively drug-resistant TB [30]. Therefore, aggressive new strategies and increased investments, and commitments to prevent, diagnose, and treat multidrug-resistant TB are required.

### Enteric infections

Despite decreasing trends from 1990 to 2019, mortality rates of enteric infections were relatively high in India. A previous study showed that diarrheal diseases were the only infectious diseases of the 3 leading individual causes of disability-adjusted life-years in India in 2016 [11]. Exposure to unsafe sanitation decreased by 43.5% and that to unsafe water source decreased by 16.9% from 1990 to 2016 in India [11] However, in 2010, the access to improved drinking water (90.7%) and the access to improved sanitation (34.2%) in India were the lowest among BRICS; and the prevalence of open defecation (50.8%) in India was much higher than other 4 countries, while the second-highest was 7.0% in South Africa [31]. These facts emphasized the importance for India of changing people’s lifestyle and continuing to improve sanitation, in order to control the transmission of diarrheal diseases.

### NTDs and malaria

India and Brazil had the highest mortality from NTDs and malaria among BRICS. NTDs, currently affecting over 1.7 billion people worldwide, referred to an entire group of tropical diseases except malaria [32]. India experienced the world’s largest absolute burden of at least 11 major NTDs [8]. The high disease-burden NTDs in India were not evenly distributed, but were instead focused in areas of urban and rural poverty (8). Since NTDs had the ability to reduce worker productivity and child intellectual growth – and ultimately impair India’s economy – it was important for the global community to focus on India’s NTD problem and make inroads [33]. Geographically, 60% of the Amazon rainforest is in Brazil, which might increase incidence of NTDs and malaria by the deforestation, proliferation of forest edges, standing water along forest margins, and many other factors [34,35]. Meanwhile, countries near Brazil, such as Colombia, Ecuador, and Venezuela, had increasing malaria incidence recent years [36]. Therefore, concerted efforts by Amazon countries are needed to avoid transnational transmission of NTDs and malaria. Currently, there are no other options besides supportive treatment and vector control for dengue and other *Aedes*-transmitted diseases, hence the need for development solutions to limit the transmission of NTDs and malaria.

### Other infectious diseases

Our results showed deaths resulting from meningitis, encephalitis, and acute hepatitis were still threats to people’s life in BRICS. India and China had the second- and third- highest ASR of Hepatitis E virus infection. However, there was no significant external funding for public health programs addressing viral hepatitis, apart from HBV vaccination [9,10]. Meningitis and encephalitis are medical emergencies that rapidly progress to death in as many as 40% of patients, while survivors may have long term deficits of neurological function [37]. Therefore, prevention for the vulnerable, as well as prompt evaluation and immediate empirical therapy for patients, are of vital importance to reduce the likelihood of fatal outcomes and chronic neurological sequelae of meningitis and encephalitis.

## CONCLUSIONS

In BRICS, despite the reductions in number of deaths from and mortality rates of infectious diseases except HIV/AIDS and STIs, there were still multiple problems to be solved. Mortality rates of respiratory infections and TB were relatively high in all BRICS countries. Brazil had the second-highest mortality from NTDs and malaria; the elderly in Russia had increased mortality rates of respiratory infections and TB; mortality rates of all kinds of infectious diseases except HIV/AIDS and STIs were relatively high in India, especially for enteric infections in middle and older aged groups; China had a still increasing mortality rate of HIV/AIDS and STIs in 2019; and South Africa had the largest number of deaths from and highest mortality rates of HIV/AIDS and STIs. Mortality from infectious diseases varies substantially in BRICS, and health disparity needs to be considered when facing complex infectious disease situations in different countries.
